# Neuropsychological and psychiatric outcomes in encephalitis: A multi-centre case-control study

**DOI:** 10.1371/journal.pone.0230436

**Published:** 2020-03-25

**Authors:** Lara Harris, Julia Griem, Alison Gummery, Laura Marsh, Sylviane Defres, Maneesh Bhojak, Kumar Das, Ava Easton, Tom Solomon, Michael Kopelman, on behalf of ENCEPH UK study group

**Affiliations:** 1 Institute of Psychiatry, Psychology and Neuroscience, King’s College London (KCL), Camberwell, London, United Kingdom; 2 National Institute for Health Research Health Protection Research Unit in Emerging and Zoonotic Infections and Institute of Infection and Global Health, University of Liverpool, Liverpool, United Kingdom; 3 Tropical and Infectious Diseases Unit, Royal Liverpool and Broadgreen University Hospitals NHS Trust, Liverpool, United Kingdom; 4 The Walton Centre NHS Foundation Trust, Liverpool, United Kingdom; 5 Encephalitis Society, Malton, United Kingdom; 6 Institute of Infection and Global Health, University of Liverpool, Liverpool, United Kingdom; Nathan S Kline Institute, UNITED STATES

## Abstract

**Objectives:**

Our aim was to compare neuropsychological and psychiatric outcomes across three encephalitis aetiological groups: Herpes simplex virus (HSV), other infections or autoimmune causes (Other), and encephalitis of unknown cause (Unknown).

**Methods:**

Patients recruited from NHS hospitals underwent neuropsychological and psychiatric assessment in the short-term (4 months post-discharge), medium-term (9–12 months after the first assessment), and long-term (>1-year). Healthy control subjects were recruited from the general population and completed the same assessments.

**Results:**

Patients with HSV were most severely impaired on anterograde and retrograde memory tasks. In the short-term, they also showed executive, IQ, and naming deficits, which resolved in the long-term. Patients with Other or Unknown causes of encephalitis showed moderate memory impairments, but no significant impairment on executive tests. Memory impairment was associated with hippocampal/medial temporal damage on magnetic resonance imaging (MRI), and naming impairment with left temporal and left frontal abnormalities. Patients reported more subjective cognitive complaints than healthy controls, with tiredness a significant problem, and there were high rates of depression and anxiety in the HSV and the Other encephalitis groups. These subjective, self-reported complaints, depression, and anxiety persisted even after objectively measured neuropsychological performance had improved.

**Conclusions:**

Neuropsychological and psychiatric outcomes after encephalitis vary according to aetiology. Memory and naming are severely affected in HSV, and less so in other forms. Neuropsychological functioning improves over time, particularly in those with more severe short-term impairments, but subjective cognitive complaints, depression, and anxiety persist, and should be addressed in rehabilitation programmes.

## Introduction

Encephalitis–which refers to inflammation of the brain caused by infection or autoimmune disease–can have debilitating long-term consequences [1]. Infectious causes, in particular herpes simplex virus (HSV), have long been considered the main cause of encephalitis [2,3]. The relatively recent discovery of cell-surface antibodies has enabled a broadening of the search for aetiologies of encephalitis. This has allowed the accurate diagnosis of an increasing number of patients with autoimmune encephalitis [4,5], including those involving the n-methyl-d-aspartate (NMDA) receptor [6] and the leucine-rich glioma 1 **(**LGI-1) protein [7]. However in 30–50% of encephalitis patients, the aetiology still remains unknown, despite intensive investigations [2,3,8,9].

There have been few studies comparing neuropsychological outcomes across different encephalitis aetiologies. Before the identification of cell-surface antibodies, several studies were published on outcomes in patients recovering from HSV encephalitis [10–12]. Compared with other infectious aetiologies, Hokkanen et al. [13] found that HSV patients showed greater difficulty on verbal memory, semantic, and visuo-spatial functions than the other aetiological groups. Studies focusing specifically on infectious causes of encephalitis other than HSV found impairments in executive function [14], retrograde memory [13], visuo-spatial processing [15], and working memory [16]. Pewter et al. [17] compared HSV and ‘non-HSV’ patients, who included infectious and autoimmune aetiologies. They found significant and widespread cognitive deficits in HSV patients, whereas ‘non-HSV’ patients showed more isolated disorders of executive function [17]. Research focusing on autoimmune aetiologies indicates neuropsychological impairments in attention, executive function, working memory, visuospatial and language abilities, and memory [18–21]. However, these various studies did not use uniform neuropsychological assessments to measure outcome, nor did they investigate how outcomes related to patients’ self-reported perception of their cognitive abilities, depression, or anxiety. In most cases, they also did not examine changes in outcomes over time.

In terms of neuroimaging, HSV encephalitis causes persisting hyper-intensities on T2-weighted MRI scans and substantial volume loss in the medial temporal lobes [10,22]. Lateral temporal and widespread cortical damage is also common [10,22,23]. The severity of anterograde amnesia after HSV encephalitis appears to be correlated with the extent of pathology in the medial temporal regions, with bilateral damage predictive of severe amnesia [10,11]. Limbic encephalitis with autoimmune aetiologies has been specifically associated with hippocampal atrophy, which is reported to correlate with disease severity and memory deficits [22–24].

In this study, we have investigated (i) neuropsychological outcomes; (ii) patients’ perceptions of their cognition, and (iii) psychiatric outcomes in terms of depression and anxiety at three time-points after hospital discharge. We examined how this was related to clinical variables and neuroimaging findings. The study was a component of a NIHR programme grant on encephalitis (ENCEPH-UK, www.encephuk.org), examining predictors of outcome [25,26].

### Aims

The purpose of the current study was to examine:

1. The presence of distinct patterns of neuropsychological impairment across different subgroups of encephalitis patients, e.g. whether memory impairments were more evident in specific diagnostic subgroups, whether executive function impairments were more pronounced in other causes of encephalitis, and whether there were differences in IQ and naming;
2. The patients’ perceptions of the impact of encephalitis on their lives, with respect to subjective, self-reported cognitive complaints, depression and anxiety;
3. The changes in neuropsychological performance across encephalitis subgroups when measured at different time-points; and
4. The relationship between clinical variables, neuroimaging, and neuropsychological/psychiatric outcomes.

## Methods

**Fig 1:**
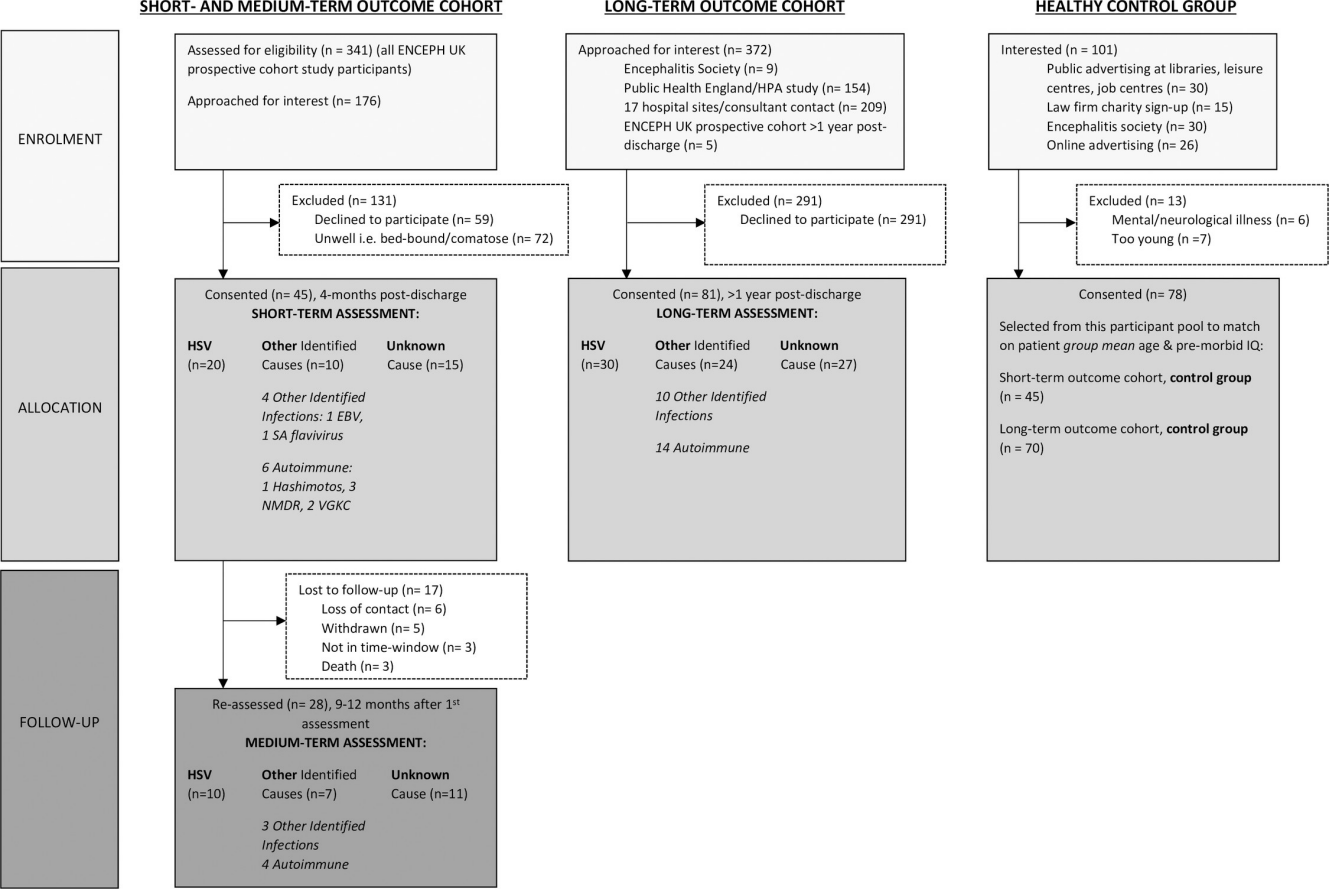
Flowchart to outline the details of the recruitment process of patients with encephalitis into the short- and medium-term and long-term outcome cohorts, and of healthy controls.

Two cohorts of encephalitis patients were recruited and investigated between 2012 and 2015. The first cohort was assessed 4 months after their hospital discharge to measure ‘short-term’ outcomes, and then again 9–12 months after the first assessment to examine ‘medium-term’ outcomes. This will be referred to as the “short- and medium-term outcome cohort”. The second cohort was recruited at least 1 year after hospital discharge in order to examine the ‘long-term’ outcomes. This will be referred to as the “long-term outcome cohort”. In both cohorts, patients with current or prior neurological conditions or brain injuries impacting on cognitive function were excluded in order to ascertain the specific association between neuropsychological impairment and encephalitis. Migraine was not an exclusion criterion. A healthy control group without neurological or psychiatric illness, subsequently referred to as “healthy controls” or “healthy control group”, was recruited from the public. Fig 1 shows the recruitment details of both patient cohorts as well as the healthy control group. The study received ethical approval by the NRES Committee East Midlands–Nottingham 1 REC (ref 11/EM/0442). Written informed consent was obtained from all participants.

For the main analyses, patients in each cohort were categorised into three diagnostic groups: (i) HSV encephalitis (referred to as ‘HSV’), (ii) Encephalitis of Other Identified Causes (referred to as ‘Other’), and (iii) Encephalitis of Unknown Cause (referred to as ‘Unknown’). This was done according to predetermined case definitions for HSV and other infectious causes [25–27] and autoimmune encephalitis [28]. Medical notes and discharge letters were additionally reviewed by the study’s panel of infectious disease and neurology specialists.

For further exploratory analyses, the Other group was split into two subgroups: encephalitis resulting from non-HSV infection (referred to as ‘Infections’) and autoimmune encephalitis (referred to as ‘Autoimmune’).

### Participants

#### Short- and medium-term outcome cohort

Patients in the short- and medium-term outcome cohort were identified by research nurses on hospital wards at ENCEPH UK sites. Forty-five participants with encephalitis were recruited and assessed approximately 4 months after hospital discharge (4.3±1.9 months) to assess short-term outcomes. When available, they were assessed again 9–12 months after the first assessment (*N* = 28, 10.5±1.9 months) for medium-term outcomes. There were 20 patients included in the HSV group, 10 patients in the Other group, and 15 patients in the Unknown group. Table 1 shows the means, standard deviations and frequencies for age, gender and Glasgow Coma Scale (GCS) scores at admission across these three diagnostic groups.

**Table 1:** Demographic characteristics of patients and controls in the short- and medium-term outcome cohort as well as patients and controls in the long-term outcome cohort.

	HSV Encephalitis (n = 20)	Encephalitis of Other named cause (n = 10)	Encephalitis of Unknown cause (n = 15)	Healthy Controls (n = 45)	Test statistic	p-value
**Short- and medium-term outcome cohort**						
Age, years (SD)	53.05 (13.93)	46.00 (16.73)	53.40 (13.51)	51.16 (16.82)	*F* = 0.55	0.648
Male, n (%)	11 (55%)	3 (30%)	8 (53%)	23 (51%)	*X*^2^ = 1.89	0.596
GCS at admission	13.53 (1.46)	13.63 (1.41)	13.00 (2.45)	-	*F* = 0.41	0.667
**Long-term outcome cohort**						
Age, years (SD)	48.37 (13.80)	46.50 (17.93)	50.00 (12.56)	50.80 (16.91)	*F* = 0.506	0.679
Male, n (%)	10 (33%)	12 (50%)	11 (41%)	32 (46%)	*X*^*2*^ = 1.89	0.596

The means (SD) and frequencies of demographic characteristics of the participants in this study. Statistical analyses reported here include ANOVA (*F*) and Chi-Squared Test of Independence (*X*^*2*^). GCS score at admission was not available for the long-term outcome cohort. *Abbreviations*: *SD (Standard Deviation)*, *GCS (Glasgow Coma Scale)*.

As mentioned above, the Other group was split into two subgroups for further exploratory analyses. In the short- and medium-term cohort, the Other group thus comprised 4 ‘Infections’ patients and 6 ‘Autoimmune’ patients.

#### Long-term outcome cohort

Patients in the long-term outcome cohort were identified by consultants across ENCEPH UK hospital sites, from the Public Health England study database [2] or they self-referred in response to a notice by the Encephalitis Society [27]. There were 81 encephalitis patients, consisting of (i) 30 HSV patients, (ii) 24 Other patients, and (iii) 27 Unknown patients (see Table 1 for the means, standard deviations and frequencies of age and gender).

For further exploratory analyses, the Other group in this long-term outcome cohort comprised 10 ‘Infections’ patients and 14 ‘Autoimmune’ patients.

#### Healthy controls

Healthy controls without neurological or psychiatric history were recruited via community and website advertisements. From responders to these advertisements, we recruited and assessed a total of 78 participants. Based on whole-group demographic variables (mean age, gender distribution, and mean premorbid IQ), 45 healthy controls were matched to the short- and medium-term outcome cohort and 70 healthy controls were matched to the long-term outcome cohort. Table 1 shows the means, standard deviations and frequencies for age and gender in each matched healthy control group.

### Materials

Well-validated neuropsychological tests were used to assess a range of neuropsychological functions:

*Intelligence (IQ)*: *Premorbid*: Wechsler Test of Adult Reading: WTAR [29]; *Current*: Wechsler Abbreviated Scale of Intelligence, 2^nd^ edition: WASI-II [30].

*Anterograde memory*: Doors and People battery [31], including verbal and visual, recall and recognition tests.

*Retrograde memory*: Autobiographical Memory Interview [AMI, [32]], assessing personal semantic and autobiographical incidents memory in terms of total scores.

*Executive function*: Verbal fluency: FAS [33]; executive control: Trail Making Test [34]; response initiation/suppression speed: Hayling Test; rule detection: Brixton Test [35].

*Language*: naming: Graded Naming Test [36]; semantic access from pictures: Pyramids and Palm Trees: PPT [37].

*Perception*: Visual Object and Space Perception (VOSP) battery [38]; Benton Facial Recognition test [39].

Subjective self-report measures were used to assess psychiatric outcomes in terms of depression and anxiety, and self-perceived cognitive complaints:

*Psychiatric measures*: Beck Depression Inventory: BDI [40]; Beck Anxiety Inventory: BAI [41];

*Self-perceived cognitive complaints*: The A-B Neuropsychological Assessment (self-report) Schedule: ABNAS [42] which examines tiredness, mental speed, memory, concentration, motor skills, and language.

Information on clinical variables was also collected in the short- and medium-term outcome cohort:

Time between hospital admission and commencement of appropriate drug treatment (antiviral, immunosuppressant), duration of hospital stay, and time between hospital admission and neuropsychological testing. This information was not consistently available in the (retrospectively collected) long-term outcome cohort.

### Procedure

Neuropsychological testing was conducted at the participant’s home or one of the research sites (King’s College London, or Walton Centre, Liverpool).

The same battery of neuropsychological tests was used in the short- and medium-term outcome cohort as well as the long-term outcome cohort.

### Imaging

In the short- and medium-term outcome cohort, MRI scans were obtained during the course of clinical care [27]; the scan closest in date to the first neuropsychological assessment was analysed.

MRI analysis involved a qualitative, systematic assessment by two neuroradiologists. They knew the patients had encephalitis but were blinded to aetiology and neuropsychological test results. The brain was evaluated in pre-specified regions-of-interest: hippocampi (head, body and tail), amygdalae, para-hippocampal gyri, occipito-temporal gyri, lateral temporal lobes (temporal stem, superior, middle and inferior temporal gyri), insulae, cingulate gyri, fornices, mammillary bodies, and frontal lobes (inferior and dorsolateral areas). These regions were examined for oedema (signal change on T2-weighted or fluid-attenuated inversion recovery [FLAIR] images), parenchymal loss (including cystic encephalomalacia), or gliosis. We focused on the site of damage, rather than its nature (inflammatory change, signal alteration, volume loss), and we determined whether ‘damage’ was present or absent at each anatomical location. The inter-rater reliability between the two neuroradiologists was assessed using a Kappa coefficient score on this binary evaluation (presence/absence of damage) across all brain regions.

### Statistical analysis

Shapiro-Wilk tests were used to assess for normality. One-way ANOVAs (parametric) or Kruskal-Wallis tests (non-parametric) were conducted. ANCOVAs were used when neuropsychological scores correlated significantly with either BDI or BAI. For parametric data, Hochberg GT2 (homogeneity assumed) or Games-Howell (homogeneity not assumed) post-hoc tests were applied to compare performance in individual encephalitis groups with that of healthy controls. For non-parametric data, Mann-Whitney U tests were used for pairwise comparisons; here, a corrected alpha of *p* = 0.02 was applied. In the short- and medium-term outcome cohort, Spearman correlations were used to analyse associations between clinical and neuropsychological variables, and where these were significant, linear regression analyses were conducted, which included age and premorbid IQ as confounding variables. To increase the sample size, all patients were merged into one group for correlation and regression analyses.

As described above, the Other group in both cohorts was subdivided into other Infections and Autoimmune aetiological subgroups. Thus, for the further exploratory analyses there were a total of four patient groups in each cohort: HSV, Infections, Autoimmune, and Unknown. Each of these four patient groups per cohort was compared with its own healthy control group, matched for mean age, gender distribution, and mean premorbid IQ. T-tests or Mann-Whitney U comparisons were then employed to identify specific neuropsychological impairments within these aetiologies.

Rate of change between short- and medium-term timepoints was analysed by conducting one-way ANOVAs or Kruskal-Wallis tests on difference scores. These analyses were conducted only in the 28 participants who were assessed at both timepoints. Those lost to follow-up were not included.

In the short- and medium-term outcome cohort, imaging (damage/no damage) comparisons between groups were analysed using chi-square test of independence, and associations between imaging and neuropsychological findings were examined using point-biserial correlations. Where correlations were significant, linear regression analyses were conducted and included age and premorbid IQ as confounding variables.

## Results

### Short- and medium-term outcome cohort

To examine short-term outcomes, 45 patients were assessed and compared with 45 healthy controls (see Table 1 for demographic characteristics and Table 2 for neuropsychological/psychiatric outcome means and standard deviations). The recruitment flowchart outlines the specific aetiologies within each patient group (Fig 1).

**Table 2:** Short-term neuropsychological and psychiatric assessment scores in the short- and medium-term outcome cohort.

		HSV (n = 20)	Other (n = 10)	Unknown (n = 15)	Healthy Controls (n = 45)	Test statistic	*p*-value
**Intelligence (WASI)**	Fullscale IQ	93.61 (12.86) **†	90.90 (12.05) *†	106.60 (17.25)	105.87 (11.98)	*F* = 3.41 ♦□	0.020
	Verbal IQ	94.25 (12.95)	88.13 (11.48) *▪	103.07 (17.37)	105.14 (11.25)	*F =* 3.83 ♦□	0.010
	Performance IQ	96.19 (13.29)	95.50 (13.25)	108.36 (17.64)	105.95 (13.96)	*F* = 1.47 ♦	0.230
**Retrograde memory (AMI)**	Personal semantic	50.29 (6.93) **◊	57.44 (4.44)	55.92 (7.21)	59.27 (4.18)	*H* = 23.46	<0.001
	Autobiographical incidents	16.97 (5.70) **◊	17.56 (4.50) **◊	19.12 (8.02)	23.32 (2.71)	*H* = 23.81	<0.001
**Executive function**	Verbal fluency ⁰ (FAS)	38.11 (14.90) **†	48.40 (9.18)	48.71 (24.38)	50.95 (13.08)	*H* = 10.91	0.010
	Executive Control ⁰ (Trails A-B)	74.21 (89.51)	42.53 (24.20)	39.43 (40.58)	39.64 (27.06)	*H* = 2.83	0.420
	Response initiation speed (Hayling)	4.83 (1.38)	5.10 (1.20)	5.43 (1.22)	5.31 (1.08)	*H* = 2.48	0.480
	Response suppression speed (Hayling)	4.44 (1.58) **◊	5.00 (2.26)	4.86 (1.70)	5.64 (0.08)	*H* = 10.52	0.020
	Response suppression accuracy (Hayling)	4.44 (2.15) **◊	6.78 (0.97)	5.50 (2.82)	6.27 (1.86)	*H* = 9.12	0.030
	Rule detection (Brixton errors)	19.72 (8.09)	20.50 (9.76)	14.21 (6.62)	15.60 (7.20)	*F* = 1.00 ♦	0.320
**Language & semantic ability**	Naming (Graded)	14.11 (6.90) **†	16.10 (5.02)	20.80 (5.51)	20.27 (5.06)	*F* = 6.93	<0.001
	Visual semantic access ⁰ (PPT)	50.17 (1.54)	50.40 (3.06)	51.77 (0.44)	50.22 (1.68)	*H* = 17.68	0.001
**Perception**	Incomplete letters ⁰ (VOSP)	19.22 (1.17)	19.80 (0.42)	19.67 (0.62)	19.43 (0.66)	*H* = 3.86	0.280
	Object decision ⁰ (VOSP)	17.22 (3.00)	18.90 (0.99)	19.87 (0.52)	17.41 (1.96)	*H* = 27.03	<0.001
	Position discrimination ⁰ (VOSP)	19.33 (1.68)	19.60 (0.70)	19.93 (0.26)	19.68 (0.67)	*H* = 2.93	0.400
	Face recognition ⁰ (Benton)	45.33 (5.11)	47.80 (2.70)	47.07 (3.45)	49.07 (3.47)	*F* = 1.63 ♦	0.190
**Psychiatric measures**	BDI	16.76 (12.41) **◊	12.3 (9.52) **◊	14.43 (10.91)	6.31 (5.89)	*H* = 16.18	0.001
	BAI	12.12 (12.51)^	9.50 (11.53)	12.21 (10.51) *◊	4.83 (5.00)	*H* = 9.18	0.030

The mean scores (standard deviations) of each patient group and the healthy control group for all neuropsychological assessments and psychiatric measures are shown in this table. Alongside are the test statistics [Kruskal-Wallis test (H); One-way ANOVA (F); or ANCOVA (F)] and all main effect p-values. For significant main effects, post-hoc tests were conducted for each patient group vs the control group and significant differences are indicated by * for p≤ 0.05 and ** for p≤ 0.01.

Notations: ⁰ all mean scores in non-impaired range

non-significant trend

† Hochberg GT2/Games-Howell post-hoc (parametric)

◊ Mann Whitney U post-hoc (non-parametric) (alpha value p = 0.02)

▪ Bonferroni-corrected pairwise analysis (ANCOVA post-hoc). ANCOVA covariates

♦ Beck Depression Inventory

□ Beck Anxiety Inventory. Abbreviations: WASI (Wechsler Abbreviated Scale of Intelligence), AMI (Autobiographical Memory Inventory), FAS (F-A-S verbal fluency test), PPT (Pyramids & Palm Trees), VOSP (Visual Object and Space Perception), BDI (Beck’s Depression Inventory), BAI (Beck’s Anxiety Inventory)

For further exploratory analyses the Other group was split into two subgroups. The 4 ‘Infections’ patients comprised 2 males and 2 females and had a mean age of 59.90 (SD = 8.81). The 6 ‘Autoimmune’ patients comprised 1 male and 5 females and had mean age of 37.00 (SD = 14.64).

### Intelligence

There was a lower mean full-scale IQ (FSIQ) in the HSV and Other groups compared with healthy controls [*F*(3, 74) = 3.41, *p* = 0.02; post-hocs *p*<0.01 and *p*<0.05, respectively]. Verbal IQ (VIQ) also differed across groups [*F*(3, 74) = 3.83, *p* = 0.01] with impairment in the Other group (post-hoc *p* = 0.02). Performance IQ (PIQ) did not differ across groups (*p* = 0.23).

### Anterograde memory

**Fig 2:**
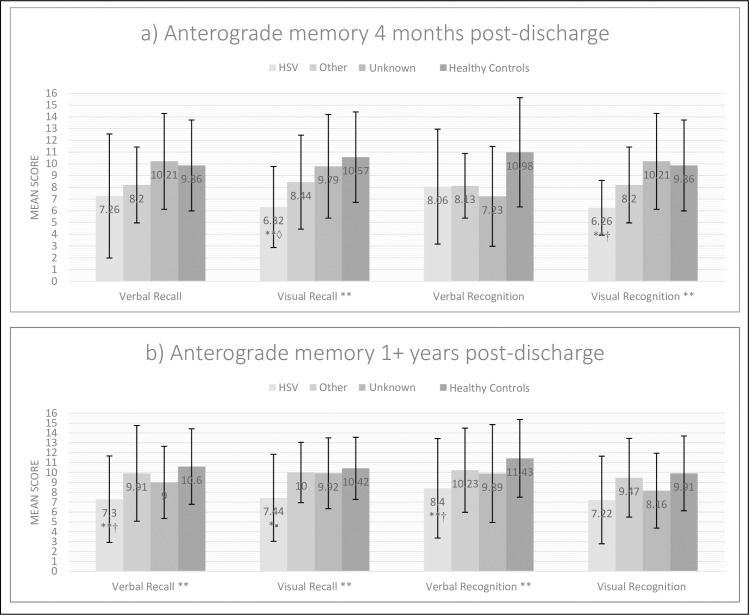
Mean anterograde memory scores for each aetiological group (Doors & People test). The error bars represent the standard deviation of the mean score within each group. (a) At the short-term assessment, a mean of 4 months post-discharge (in the short- and medium-term outcome cohort) and (b) at the long-term assessment, at least 1 year post-discharge (in the long-term outcome cohort). Notations: *p≤0.05, **p≤0.01; † Hochberg GT2/Games-Howell post hoc (parametric); ▪Bonferroni-corrected pairwise analysis (ANCOVA post hoc). ◊ Mann Whitney U post hoc (non-parametric).

Fig 2(A) shows performance on verbal and visual, recall and recognition memory. There were significant differences on visual recall [*H*(3) = 14.05, *p* = 0.003] and visual recognition memory [*F*(3, 83) = 5.54, *p* = 0.002], with significant impairments in HSV versus healthy controls on post-hoc testing (p<0.01).

### Retrograde memory

Personal semantic memory [*H*(3) = 23.46, *p*<0.001] and autobiographical incidents memory [*H*(3) = 23.81, *p*<0.001] differed across the groups (Table 2) with striking impairments in the HSV group on both components (p<0.001), and in the Other group on autobiographical incidents (*p*<0.001).

### Executive function

Verbal fluency was impaired in HSV [*H*(3) = 10.91, *p* = 0.01; post-hoc *p*<0.01]. There were significant group effects on response suppression speed [*H*(3) = 10.52, *p* = 0.02] and accuracy [*H*(3) = 9.12, *p* = 0.03], resulting from impairment in the HSV group (post-hoc p’s≤0.01).

### Language & visual semantic access

On Graded Naming, the HSV group showed severe impairment [*F*(3, 85) = 6.93, *p*<0.001; post-hoc versus healthy controls, *p*<0.01]. On visual semantic access, all mean scores were in the normal range.

### Perception

All patient groups scored within the non-impaired range and Kruskal-Wallis and ANOVA analyses did not reveal significant differences (p’s>0.19, Table 2).

### Psychiatric measures

The HSV and Other groups reported raised levels of depression [*H*(3) = 16.18, *p* = 0.001; post-hoc versus healthy controls *p*’s<0.01], and the Unknown group higher levels of anxiety than healthy controls [*H*(3) = 9.18, *p* = 0.03; post-hoc *p*<0.05] (Table 2).

### Patient-perceived (subjective) cognitive function

**Fig 3:**
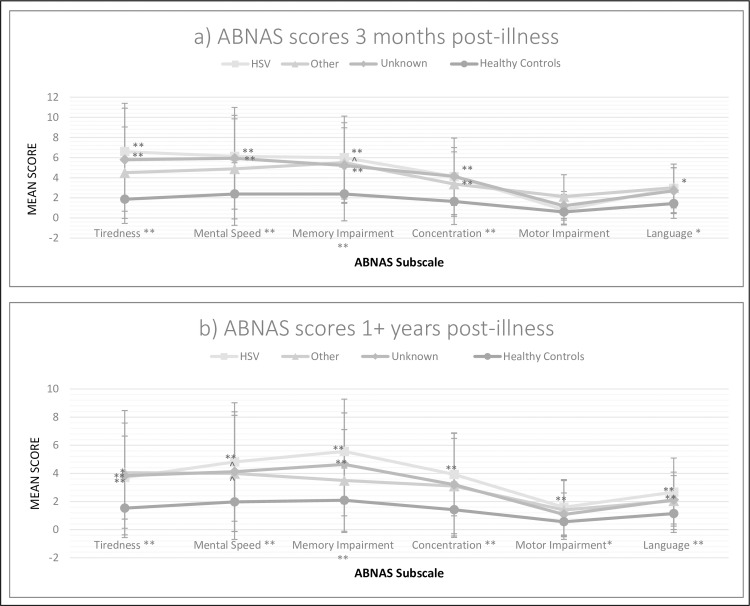
Subjective cognitive complaints for each group (ABNAS subscale scores). The error bars represent the standard deviation of the mean score within each group. (a) At the short-term assessment, a mean of 4 months post-discharge (in the short- and medium-term outcome cohort) and (b) at the long-term assessment, at least 1-year post-discharge (in the long-term outcome cohort). Notations: For main analyses of subscales (all Kruskal Wallis), significance is labelled adjacent to the subscale name along the x-axis, alpha p = 0.05, * p ≤ 0.05, ** p ≤ 0.01; For the breakdown of main analyses using Mann Whitney U post hoc, significance is labelled adjacent to data points within the figure, corrected alpha p = 0.02, * p ≤ 0.02, ** p ≤ 0.01, ^ trending significance (p = 0.03); Abbreviations: ABNAS (A-B Neuropsychological Assessment Schedule).

Fig 3(A) shows mean ratings for each group on the six ABNAS subscales. Patients reported significantly more perceived tiredness [*H*(3) = 20.37, *p*<0.001], slower mental speed [*H*(3) = 15.89, *p* = 0.001], and complaints of impairments in memory [*H*(3) = 16.05, *p* = 0.001], concentration [*H*(3) = 14.64, *p* = 0.002], and language [*H*(3) = 9.09, *p* = 0.03]. The HSV and Unknown groups reported high rates of complaints on 4 of the 6 ABNAS subscales. Correlations with objective test performance were generally weak and non-significant, with the exception of a significant correlation between ABNAS Memory and verbal recognition memory (rho = -0.35, p = 0.002).

### Rate of change between short- and medium-term outcome

To examine change between short- and medium-term outcomes, 28 of the 45 patients studied in the short- and medium-term outcome cohort were reassessed 9–12 months after their first assessment. Difference scores (T2-T1) indicating rate of change were compared across encephalitis groups.

On VIQ, there was a significant group difference in change scores [*F*(2, 24) = 4.98, *p* = 0.02]. The Other group (8.43±6.90) improved significantly more than the Unknown group (-4.27±9.77), whose score declined. There were no other significant group effects in change scores on cognitive measures (*p*’s>0.07), patient-perceived cognitive impairments (*p*’s>0.09), or mood assessments (*p*’s>0.05).

We compared mean age, GCS score at hospital admission as an indicator of illness severity, and aetiology in the participants who dropped out versus those who did not. T-tests revealed no significant differences in age or admission GCS score (p’s > 0.60). A Fisher’s exact test showed no significant differences in aetiology (p = 0.10). The reasons for drop-out most commonly cited by our participants were tiredness and physical illness.

### Clinical correlations

Table 3 shows that the time between hospital admission and commencement of appropriate treatment correlated negatively with measures of intelligence, retrograde and anterograde memory, and executive function (i.e. longer time until treatment was associated with worse performance). Older age was correlated with lower scores on naming (rho = -0.30), and more errors on the Hayling test (rho = 0.44) (both p<0.05). Treatment was instigated earlier if the GCS score was low (rho = 0.34, p<0.05). To further examine these correlations, we also carried out linear regression analyses, including time until treatment, premorbid IQ and age as potential predictors of neuropsychological outcome. On inputting these variables, we obtained significant overall regressions in predicting verbal IQ [R^2^ = .42, F(3, 37) = 8.95, p < .001], verbal recall [R^2^ = .34, F(3, 37) = 6.28, p = .001] and rule detection [R^2^ = .42, F(3, 36) = 8.69, p < .001] scores. However, of our specific predictors, only premorbid IQ (β = .60, p < .001; β = .54, p = .001; β = -.54, p < .001, respectively) and age (for rule detection only: β = .34, p = .01) were statistically significant. Time until appropriate treatment did not contribute significantly to the regression.

**Table 3:** The significant correlations between clinical variables and short-term neuropsychological assessment scores in the short- and medium-term outcome cohort.

Time since admission (months)		Time until treatment (days)		Length of stay in hospital (days)	
*Assessment*	*Statistic*	*Assessment*	*Statistic*	*Assessment*	*Statistic*
Executive control (TMT A-B)	*0*.38*	Verbal IQ	*-0*.32*	Visual recall	*-0*.41**
		Verbal recall	*-0*.36*	Response suppression accuracy	*-0*.33*
		Verbal recognition	*-0*.47**	Executive control (TMT A-B)	*0*.36*
		Rule detection	*-0*.35*	ABNAS motor impairment	*-0*.33*
				ABNAS language	*-0*.42**

Only significant correlations as assessed by Spearman rho test are shown. All patients were combined to increase the sample size.

Notations

* p ≤ 0.05

** p ≤ 0.01; Abbreviations: TMT (Trail Making Test), ABNAS (A-B Neuropsychological Assessment Schedule)

Table 3 shows that length of hospital stay also correlated significantly with measures of memory and executive function (i.e. longer stay associated with worse performance). On the other hand, it correlated with fewer patient-perceived language and motor complaints. On linear regression, increased length of stay remained a significant predictor of reduced patient-perceived language complaints when covarying for premorbid IQ and age (β = -.55, p < .001) [R^2^ = .39, F(3, 34) = 7.36, p = .001].

*Imaging*. Table 4 summarises the imaging findings on clinical MRI scans obtained closest to the time of the first neuropsychological assessment. The kappa coefficient score for inter-rater reliability between the two individuals assessing the binary presence/absence of damage was 0.98. There were significant differences between the three patient groups for hippocampal damage (particularly on the left) and medial temporal damage; both were most common in the HSV group. In contrast, dorsolateral frontal damage was most common in the Unknown group, and there were significant differences in comparing the distribution of left or right medial temporal versus dorsolateral frontal changes across groups (left, *X*^2^ = 19.21, *p*<0.001; right *X*^2^ = 19.18, *p*<0.001).

**Table 4:** Prevalence (%) of damage across brain regions in each encephalitis group on MRI.

		HSV	Other	Unknown	χ^2^
**Total patients**	** **	16	11	18	
**N scanned**	** **	14	9	14	
		**% of scanned patients with damage**			
**Hippocampi**	Left	78.6	55.6	28.6	7.06*
	Right	71.4	55.6	35.7	3.61
	Either	92.9	66.7	35.7	10.05**
**Medial temporal lobes**	Left	78.6	66.7	35.7	5.59
	Right	71.4	66.7	35.7	4.11
	Either	92.9	77.8	42.9	8.70**
**Lateral temporal lobes**	Left	35.7	33.3	21.4	0.76
	Right	35.7	33.3	14.3	1.87
	Either	71.4	44.4	28.6	5.23
**Inferior frontal lobes**	Left	28.6	22.2	7.1	2.18
	Right	21.4	33.3	14.3	1.17
	Either	42.9	33.3	14.3	2.81
**Dorsolateral frontal cortices**	Left	0.0	11.1	21.4	3.34
	Right	0.0	11.1	14.3	2.06
	Either	0.0	11.1	21.4	3.34

The percentages of patients with left-sided, right-sided or either-sided damage in hippocampi, medial temporal lobes, lateral temporal lobes, inferior frontal lobes, and dorsolateral frontal cortices is shown. Chi-squared test of independence statistics are also shown. N was determined by the number of patients with scans made available to the study researchers.

Notations

* p ≤ 0.05

** p ≤ 0.01

Table 5 shows that left hippocampal and left medial temporal damage were correlated with lower IQ; and hippocampal and medial temporal damage with lower visual recall memory as well as with poorer response initiation and suppression. Right hippocampal, medial temporal, and lateral temporal damage were all correlated with poor visual recognition memory. There were also significant correlations between left temporal or frontal damage and graded naming.

**Table 5:** The relationship between MRI findings and short-term neuropsychological outcomes.

		Intelligence			Memory				Language & semantic ability	Executive function					
		Verbal IQ	Performance IQ	Fullscale IQ	Visual recall	Verbal recall	Visual recognition	Verbal recognition	Graded naming (total correct)	FAS	Executive control (TMT A-B)	Response initiation speed (Hayling)	Response suppression speed (Hayling)	Response suppression accuracy (Hayling)	Rule detection (Brixton)
**Hippocampus**	**Left**	-0.29	-0.32	-0.34*	-0.35*	-0.23	-0.25	-0.08	-0.29	-0.30	0.16	-0.41*	-0.20	-0.43**	-0.11
	**Right**	-0.24	-0.31	-0.30	-0.43**	-0.17	-0.38*	0.00	-0.16	-0.20	0.15	-0.37*	-0.44**	-0.23	-0.06
**Medial temporal lobe**	**Left**	-0.35*	-0.33*	-0.38*	-0.38*	-0.26	-0.21	-0.20	-0.34*	-0.29	0.14	-0.41*	-0.27	-0.36*	-0.18
	**Right**	-0.25	-0.31	-0.31	-0.51**	-0.12	-0.43**	-0.01	-0.16	-0.13	0.15	-0.28	-0.40*	-0.19	-0.16
**Lateral temporal lobe**	**Left**	-0.24	-0.02	-0.16	0.05	-0.12	0.08	-0.31	-0.39*	-0.24	-0.14	-0.25	-0.07	-0.25	-0.09
	**Right**	-0.24	-0.29	-0.29	-0.18	-0.03	-0.34*	0.03	-0.14	-0.07	-0.08	-0.15	-0.18	-0.08	-0.01
**Inferior frontal lobe**	**Left**	-0.30	-0.25	-0.31	-0.12	-0.12	-0.13	0.00	-0.35*	-0.13	-0.07	-0.06	0.04	-0.21	-0.07
	**Right**	-0.17	-0.26	-0.23	-0.15	0.05	-0.31	0.15	-0.05	0.00	-0.06	0.16	-0.24	-0.02	-0.12
**Dorso-lateral frontal cortex**	**Left**	0.14	0.04	0.10	0.24	0.02	0.21	-0.03	0.11	-0.10	-0.06	0.14	0.16	0.12	0.14
	**Right**	0.07	-0.11	-0.02	0.24	-0.03	0.09	0.12	0.07	-0.17	-0.03	0.08	0.09	0.02	0.12

Point-biserial correlations between the presence or absence of damage and neuropsychological scores are shown.

Notations

** p≤0.01

* p≤0.05. Abbreviations: TMT (Trail Making Test), FAS (F-A-S verbal fluency test)

To explore these associations further, regression analyses accounting for premorbid IQ and age revealed that left medial temporal damage significantly predicted verbal IQ [R^2^ = .56, F(3, 30) = 12.59, p < .001; β = -.26, p = .05], fullscale IQ [R^2^ = .57, F(3, 30) = 13.27, p < .001; β = -.25, p = .05] and naming deficits [R^2^ = .48, F(3, 31) = 9.51, p < .001; β = -.27, p = .05]. Right hippocampal damage predicted lower visual recall memory [R^2^ = .28, F(3, 29) = 3.72, p = .02; β = -.34, p = .04] and right medial temporal damage significantly predicted lower visual recall memory [R^2^ = .32, F(3, 29) = 4.55, p = .01; β = -.41, p = .02] and lower visual recognition memory [R^2^ = .23, F(3, 30) = 2.93, p = .05; β = -.35, p = .04]. Finally, left lateral temporal damage significantly predicted naming impairment [R^2^ = .50, F(3, 31) = 10.12, p < .001; β = -.30, p = .03].

### Long-term outcome cohort

Eighty-one patients completed neuropsychological and psychiatric assessments more than one year after acute encephalitis and were compared with the scores of 70 healthy controls matched on age and premorbid IQ. Table 1 summarises the demographic characteristics across groups and Table 6 shows the means and standard deviations of the neuropsychological and psychiatric outcome measures.

**Table 6:** Long-term neuropsychological and psychiatric assessment scores in the long-term outcome cohort.

		HSV (n = 30)	Other (n = 24)	Unknown (n = 27)	Healthy Controls (n = 70)	Test statistic	*p*-value
**Intelligence (WASI)**	Fullscale IQ	105.07 (16.64)	108.82 (11.61)	102.74 (15.27)	108.24 (11.91)	*F* = 1.39	0.25
	Verbal IQ	101.48 (17.58)	103.35 (14.89)	100.00 (16.03)	106.71 (11.28)	*F =* 1.90	0.13
	Performance IQ	108.21 (18.71)	111.55 (11.43)	105.11 (19.29)	107.89 (14.17)	*F* = 0.67	0.57
**Retrograde Memory (AMI)**	Personal semantic	55.48 (7.24)	57.00 (5.51)	56.70 (6.37)	58.72 (4.20)	*H* = 4.88	0.18
	Autobiographical incidents	19.14 (5.45) **◊	20.90 (6.24)	20.63 (5.20) *◊	23.27 (2.65)	*H =* 13.15	0.004
**Executive function**	Verbal fluency⁰ (FAS)	49.90 (14.26)	48.84 (13.10)	47.04 (12.81)	54.01 (14.32)	*F =* 2.03	0.11
	Executive Control ⁰ (Trails A-B)	53.80 (48.46)	33.02 (29.44)	35.45 (28.50)	34.94 (24.73)	*H* = 3.61	0.31
	Response initiation speed⁰ (Hayling)	5.03 (1.75)	5.43 (1.20)	5.37 (1.04)	5.50 (0.96)	*H* = 2.53	0.31
	Response suppression speed⁰ (Hayling)	5.03 (1.56)	5.43 (1.27)	5.19 (1.21)	5.71 (0.75)	*H* = 5.96	0.11
	Response suppression accuracy⁰ (Hayling)	6.07 (2.07)	5.83 (2.69)	7.74 (2.40)	6.30 (1.81)	*H* = 0.44	0.93
	Rule detection⁰ (Brixton errors)	16.87 (7.60)	16.57 (10.06)	16.22 (7.68)	14.01 (6.57)	*H* = 3.92	0.27
**Language & semantic ability**	Naming (graded)	16.70 (7.73) ^	21.96 (4.85)	19.48 (4.79)	20.66 (4.92)	*F* = 4.01 □	0.009
	Visual semantic access⁰ (PPT)	50.17 (2.26)	51.39 (0.78)	51.52 (0.58)	50.19 (1.83)	*H* = 23.42	<0.001
**Perception**	Incomplete letters⁰ (VOSP)	19.54 (1.04)	19.32 (1.00)	19.78 (0.51)	19.38 (0.75)	*H =* 8.22	0.04
	Object decision⁰ (VOSP)	18.17 (2.24)	18.59 (1.56)	18.30 (1.88)	17.21 (2.00)	*H* = 14.81	0.002
	Position discrimination⁰ (VOSP)	19.31 (2.49)	19.86 (0.47)	19.48 (1.40)	19.68 (0.68)	*H* = 1.96	0.58
	Face recognition⁰ (Benton)	47.60 (4.17)	48.48 (4.23)	48.19 (2.29	48.74 (3.47)	*H* = 2.54	0.47
**Psychiatric measures**	BDI	13.81 (8.13) **◊	12.65 (12.15) *◊	12.20 (11.15) **◊	5.48 (5.37)	*H* = 26.01	<0.001
	BAI	10.04 (9.51) **◊	10.00 (9.80) **◊	9.36 (9.38) *◊	4.36 (4.64)	H = 13.58	0.004

The mean scores (standard deviations) of each patient group and the healthy control group for all neuropsychological assessments and psychiatric measures are shown in this table. Alongside are the test statistics [Kruskal-Wallis test (H); One-way ANOVA (F); or ANCOVA (F)] and all main effect p-values. For significant main effects, post-hoc tests were conducted for each patient group vs the healthy control group and significant differences are indicated by * for p≤ 0.05 and ** for p≤ 0.01.

Notations: ⁰ all mean scores in non-impaired range

^ non-significant trend (0.05<p<0.07)

† Hochberg GT2/Games-Howell post-hoc (parametric)

◊ Mann Whitney U post-hoc (non-parametric) (alpha value p = 0.02)

▪ Bonferroni-corrected pairwise analysis (ANCOVA post-hoc). ANCOVA covariates

♦ Beck Depression Inventory

□ Beck Anxiety Inventory. Abbreviations: WASI (Wechsler Abbreviated Scale of Intelligence), AMI (Autobiographical Memory Inventory), FAS (F-A-S verbal fluency test), PPT (Pyramids & Palm Trees), VOSP (Visual Object and Space Perception), BDI (Beck’s Depression Inventory), BAI (Beck’s Anxiety Inventory)

For further exploratory analyses, within the Other group, there were 10 ‘Infections’ patients comprising 5 males and 5 females with a mean age of 49.70 (SD = 16.17), and 14 ‘Autoimmune’ patients comprising 7 males and 7 females with a mean age of 44.21 (SD = 16.20).s

### Intelligence

No significant group differences were found on current FSIQ, VIQ, or PIQ (*p*’s>0.13).

### Anterograde memory

Fig 2(B) shows significant group effects on verbal recall [*F*(3, 143) = 4.79, *p* = 0.003], visual recall [*F*(3, 128) = 3.36, *p* = 0.02], and verbal recognition memory [*F*(3, 143) = 3.41, *p* = 0.02] with the HSV group severely impaired relative to healthy controls (post-hoc *p*’s<0.02). By contrast, there were no group differences on visual recognition memory (*p* = 0.10).

### Retrograde memory

Personal semantic memory did not differ significantly across groups (*p* = 0.18, Table 6). There was a significant group effect on autobiographical incidents memory [*H*(3) = 13.15, *p* = 0.004], reflecting impaired HSV and Unknown performance relative to healthy controls (post-hoc *p*<0.001 and *p* = 0.03, respectively).

### Executive function

Table 6 shows all executive function scores were in the non-impaired range (*p*’s>0.11).

### Language & visual semantic access

On Graded Naming, the groups differed significantly [*F*(3, 132) = 4.01, *p* = 0.009], but post-hoc analyses showed only a non-significant trend in the HSV group (*p* = 0.06, Table 6).

On visual semantic access, all scores approached ceiling.

**Perception.** Performance approached ceiling on all perception measures (Table 6).

**Psychiatric measures.** There were higher levels of depression [*H*(3) = 26.01, *p*<0.001] and anxiety [*H*(3) = 13.58, *p* = 0.004] in all patient groups, relative to healthy controls (post-hoc *p*’s<0.04) (Table 6).

**Patient-perceived (subjective) cognitive function.** Patients reported more complaints than healthy controls [Fig 3(B)] in terms of tiredness [*H*(3) = 15.88, *p* = 0.001], mental speed [*H*(3) = 13.31, *p* = 0.004], and impairments in memory [*H*(3) = 20.58, *p*<0.001], concentration [*H*(3) = 15.21, *p* = 0.002], motor ability [*H*(3) = 9.69, *p* = 0.02], and language [*H*(3) = 13.10, *p* = 0.004]. Fig 3(B) shows that the HSV group expressed significantly more complaints than healthy controls across all domains (post-hoc *p*’s<0.01). The Unknown group also reported more difficulty than healthy controls on memory, language, and tiredness (post-hoc *p*’s<0.01). The Other group reported greater tiredness than healthy controls (post-hoc *p* = 0.03).

On comparing the Autoimmune patient group with its matched healthy control group, there were more frequent complaints concerning mental speed (*U* = 24.00, *p* = 0.006), memory (*U* = 31.50, *p* = 0.02), and concentration (*U* = 32.00, *p* = 0.02).

There were some weak but statistically significant correlations between subjective (ABNAS) ratings and objective test performance: ABNAS Memory complaints correlated with (impaired) verbal and visual recall and verbal recognition memory scores (rho = -0.17 to-0.29, p<0.05), and ABNAS Language complaints with (lower) FAS scores (rho = -0.24, p = 0.005).

## Discussion

The current study investigated neuropsychological and psychiatric outcomes across three different encephalitis groups in the short-term (4 months post-discharge), medium-term (9-12-months follow-up after first assessment), and the long-term (>1 year post-discharge). We found that that neuropsychological and psychiatric outcomes after encephalitis varied according to the aetiology. Memory and naming impairments were most severely affected the HSV group, and this was associated with more severe medial temporal lobe damage on MRI. Across groups, cognitive performance improved over time, but subjective cognitive complaints, depression, and anxiety persisted. We will discuss these findings with respect to our original aims in turn.

In line with our first aim, which was to examine neuropsychological profiles across encephalitis aetiological groups, differential patterns of neuropsychological impairment were found across the patient groups. In the short-term, both HSV and patients with Other or Unknown causes of encephalitis were impaired on anterograde memory, but this was more severe in the HSV group, consistent with previous findings [15,17]. The Other group was also impaired across anterograde memory tests, statistically significant for verbal recognition memory. In terms of autobiographical memory, HSV patients showed short-term (<1 year) impairments in the recall of personal semantic facts, and short- and long-term impairments (>1 year) in recalling autobiographical episodes. The Unknown aetiology group also showed long-term impairment in recalling autobiographical memories. Other authors have also found mild/moderate memory impairments in ‘non-HSV’ encephalitis patients [13,17].

With respect to other cognitive functions, we did not find more pronounced executive impairment in either the Other or the Unknown groups than the HSV patients, contrary to Pewter et al.’s [17] finding of isolated executive impairment in ‘non-HSV’ patients. In the short-term, only the HSV group showed impairments on executive function, naming, and full-scale IQ. The naming impairment occurred in the presence of normal verbal fluency (FAS) and normal visual semantic access. In the short-term, both the Other and the Unknown groups were unimpaired on executive function and naming, and only the Other group was impaired on verbal and full-scale IQ. In the long-term, the HSV group showed only a trend towards impairment on naming, but otherwise there were no long-term impairments across the aetiological groups on executive, naming, or IQ tasks. In other words, executive function, naming, and IQ impairments appeared to resolve after the first year.

With respect to our second aim, which was to assess the patients’ subjective, self-reported perceptions of their cognitive abilities, there were differences in tiredness, mental speed, concentration, memory, and language in the encephalitis groups compared with the healthy controls. These occurred both in the short- and long-term after the illness (Fig 3), with HSV the most, and the Other group the least, severely affected. Tiredness was a particularly prominent complaint, as has also been reported elsewhere [43]. With respect to psychiatric outcomes, there were significantly higher short-term rates of depression in the HSV and Other groups, and anxiety in the Unknown group, than in healthy controls. In the Unknown group, this anxiety might reflect the negative impact of receiving an uncertain diagnosis. In the long-term, all patient groups continued to show raised levels of depression and anxiety. This is in line with findings from another study which examined psychiatric sequelae of encephalitis, suggesting depression and anxiety can arise both from neurobiological changes and from the psychological adjustment to encephalitis [44]. It is notable that, in terms of patients’ subjective perceptions of cognitive function and mood state, complaints remained severe after the first year. This appears particularly at odds with current and previous suggestions of improved objectively measured cognitive function over time [45,46], and may reflect the dawning realisation that further improvement after severe neurological illness was likely to be slow and limited [43,47–49]. Correlations between subjective complaints and objective performance were generally low.

The third aim was to examine the rate of change between the short (4 months)- and medium-term (9–12 months later) post-discharge. Rates of change were broadly equivalent across the patient groups during the first year, except that the Other group showed significant improvement on verbal IQ, whereas the Unknown group showed a decline in verbal IQ. During this first year, the HSV group showed a widespread pattern of cognitive impairment (memory, executive functioning, verbal IQ performance, and naming). After the first year, these had narrowed down to a predominant deficit in anterograde and retrograde memory. In the Other and Unknown cause groups, cognitive deficits which had been evident during the first year were no longer present, except for the Unknown group’s performance on autobiographical event memory.

Fourthly, we examined the relationship between clinical variables and short-term neuropsychological and psychiatric outcomes. Time between admission and commencement of appropriate treatment, as well as a longer hospital stay, were both correlated with poorer memory and executive outcomes, emphasizing the importance of timely treatment [2,21]. Timely treatment may be particularly important in older adults and those with lower premorbid intellectual abilities. Furthermore, despite the association with poorer memory and executive outcome on objective assessments, longer hospital stay was also correlated with fewer self-reported motor and language complaints, possibly because these had been more fully addressed during a more extensive stay. In line with this, a longer hospital stay significantly predicted lower self-reported language complaints even after accounting for age and premorbid IQ. It is possible that reducing self-reported complaints by providing thorough clinical care may improve mood, which in turn might reduce complaints further [50] and enhance objectively measured cognitive performance–this requires further investigation.

With regard to the MRI findings across all three patient groups, there were significant correlations between short-term visual recall and recognition scores and the presence of hippocampal and medial temporal lobe pathology. Naming impairment was associated with the presence of left-sided damage on MRI. Linear regression analyses accounting for premorbid IQ and age revealed that hippocampal and temporal damage remained significant predictors, directly supporting the relationship between brain lesion and short-term neuropsychological impairments.

### Study limitations

Even though the present study was multi-centre across UK-wide NHS departments, recruitment to the Other group, particularly the autoimmune aetiologies was difficult. At the time of recruitment, cell-surface antibodies aiding autoimmune diagnosis were not always consistently being tested in hospitals. In this neuropsychological sub-study, HSV cases comprised 33.3% of the short- and medium-term cohort, and 55% of the long-term cohort. The ENCEPH-UK parent study comprised 20% of HSV cases, which is very similar with the 19% HSV cases recruited in the HPA study [2]. The increased representation of HSV cases in the neuropsychological sub-study may have resulted from a response bias since participants could opt in to take part in the neuropsychological assessments if they wished. Overall, it is therefore important to state that the diagnostic proportions reported here are not reflective of true prevalence rates, especially with recent advances in diagnostic tools for autoimmune encephalitis. Secondly, there were relatively high rates of attrition between the initial assessment and the follow-up 9–12 months later. This could not be attributed to differences in aetiology, illness severity (based on GCS at admission) or age. In fact, the reasons for drop-out most commonly cited by our participants were tiredness and physical illness, consistent with subjective complaints reported across all three encephalitis subgroups. Finally, the patient participants in the short- and medium-term outcome cohort were not the same individuals as in the long-term outcome cohort. While this does not affect the interpretation of the findings within each cohort, it is nonetheless worth noting that the ‘longitudinal’ conclusions of this study should be interpreted cautiously.

### Summary and conclusions

In summary, the HSV group was the most impaired, and memory was the most severely affected cognitive function within this group. In the short- and medium-term assessments (4 months to 1-year post-discharge), the HSV group also showed deficits in executive function, verbal IQ, and naming. In the long-term (>1 year), these deficits had narrowed down to impaired anterograde and autobiographical memory. The Other and Unknown groups also showed most impairment in memory relative to other neuropsychological functions, which is contrary to some previous observations [17]. On subjective evaluations, the HSV group reported self-perceived problems in memory, language, tiredness, concentration, and mental speed, as well as depressed mood; and these were still present at >1 year. In the Other and Unknown groups, there were also patient-perceived, subjective cognitive complaints, anxiety, and depression, with tiredness being identified as a common problem. In particular, the Other group reported high rates of depression, whereas the Unknown group described anxiety, possibly indicating that receiving an uncertain diagnosis increases anxiety. In terms of associated clinical factors, delayed time until appropriate treatment, and prolonged hospital stay, were correlated with poorer outcomes across the patient groups, underlining the importance of timely treatment [1].

These findings have implications for neuropsychological rehabilitation. Outcomes for patients with encephalitis differed across the aetiological groups, and rehabilitation should be tailored to this. It is important that psychiatric conditions including depression and anxiety are treated appropriately as this may positively influence the success of neurorehabilitation for cognitive deficits [44]. Compensatory strategies, aiming to capitalize on preserved executive function, could be used to support memory [51], and self-perceived cognitive complaints, fatigue, depression, and anxiety are particularly important to address.

## Supporting information

S1 Data The supporting information provided with this manuscript contains 3 SPSS database files. These files contain the raw data. The files labelled “short- and medium-term outcome cohort and matched controls” and “long-term outcome cohort and matched controls” contain the demographic information, neuropsychological assessment scores, psychiatric assessment scores, the self-rated cognitive impairment scores and the clinical variables where appropriate. The file labelled “short- and medium-term outcome cohort_MRI data” contains the data extracted as binary damage/no damage from clinical scans made available to the researchers.
(ZIP)
